# A simple and efficient automated microvolume radiosynthesis of [^18^F]Florbetaben

**DOI:** 10.1186/s41181-020-00113-w

**Published:** 2020-12-04

**Authors:** Ksenia Lisova, Jia Wang, Philip H. Chao, R. Michael van Dam

**Affiliations:** 1 Physics & Biology in Medicine Interdepartmental Graduate Program, University of California Los Angeles, Los Angeles, CA USA; 2 Crump Institute for Molecular Imaging, University of California Los Angeles, Los Angeles, CA USA; 3 Department of Molecular & Medical Pharmacology, University of California Los Angeles, Los Angeles, CA USA; 4 Department of Bioengineering, University of California Los Angeles, Los Angeles, CA USA

**Keywords:** Microfluidics, Radiochemistry, Beta-amyloid imaging, Florbetaben, Molar activity, Droplet synthesis, Automation, Radiolysis

## Abstract

**Background:**

Current automated radiosynthesizers are generally optimized for producing large batches of PET tracers. Preclinical imaging studies, however, often require only a small portion of a regular batch, which cannot be economically produced on a conventional synthesizer. Alternative approaches are desired to produce small to moderate batches to reduce cost and the amount of reagents and radioisotope needed to produce PET tracers with high molar activity. In this work we describe the first reported microvolume method for production of [^18^F]Florbetaben for use in imaging of Alzheimer’s disease.

**Procedures:**

The microscale synthesis of [^18^F]Florbetaben was adapted from conventional-scale synthesis methods. Aqueous [^18^F]fluoride was azeotropically dried with K_2_CO_3_/K_222_ (275/383 nmol) complex prior to radiofluorination of the Boc-protected precursor (80 nmol) in 10 μL DMSO at 130 °C for 5 min. The resulting intermediate was deprotected with HCl at 90 °C for 3 min and recovered from the chip in aqueous acetonitrile solution. The crude product was purified via analytical scale HPLC and the collected fraction reformulated via solid-phase extraction using a miniature C18 cartridge.

**Results:**

Starting with 270 ± 100 MBq (*n* = 3) of [^18^F]Fluoride, the method affords formulated product with 49 ± 3% (decay-corrected) yield,> 98% radiochemical purity and a molar activity of 338 ± 55 GBq/μmol. The miniature C18 cartridge enables efficient elution with only 150 μL of ethanol which is diluted to a final volume of 1.0 mL, thus providing a sufficient concentration for in vivo imaging. The whole procedure can be completed in 55 min.

**Conclusions:**

This work describes an efficient and reliable procedure to produce [^18^F]Florbetaben in quantities sufficient for large-scale preclinical applications. This method provides very high yields and molar activities compared to reported literature methods. This method can be applied to higher starting activities with special consideration given to automation and radiolysis prevention.

## Background

Positron emission tomography (PET) is a powerful molecular imaging tool with extensive applications in disease diagnostics and drug development, among other areas. Many tracers (labeled with positron-emitting isotopes) have been developed that can bind to specific molecular targets in vivo and allow tracking of their dynamics and location throughout the whole body. For example, a number of tracers were developed to target amyloid-beta (Aβ) plaques which are correlated with the progression of Alzheimer’s disease (Hardy and Selkoe 2002), and it is believed that the high sensitivity and specificity of PET can aid in early diagnosis of dementia and its grading (Ossenkoppele et al. 2015).

N-methyl-[^11^C]2-(4′-methylaminophenyl)-6-hydroxybenzothiazole ([^11^C]PIB), a radiofluorinated thioflavin T analogue, was the first selective radiotracer for Alzheimer’s disease research and diagnostics (Klunk et al. 2004). However, the short half-life of carbon-11 (20 min) restricts the access to this probe, especially at locations not having their own cyclotron and expertise in ^11^C-radiochemistry, thus alternative tracers labeled with longer-lived isotopes (e.g. fluorine-18, t_1/2_ = 110 min) were developed. In fact, several ^18^F-labeled amyloid imaging agents have been reported, including [^18^F]Florbetapir, [^18^F]Florbetaben and [^18^F]Flutemetamol, and were approved for use in clinical practice in the United States (Yeo et al. 2015). In this work we focus on [^18^F]Florbetaben (also known by several other names: [^18^F]FBB, [^18^F]BAY94–9172, NeuraCeq, [^18^F]AV1, [^18^F]AV1/ZK), a stilbene derivative that was designed to selectively bind to Aβ plaques (Zhang et al. 2005). [^18^F]Florbetaben ([^18^F]FBB) and other similar tracers are extremely useful for studying cases of Alzheimer’s disease both in the clinic and in research. Clinically, the tracers are used for accurate dementia grading and early detection in at-risk populations. In research, these tracers are useful for testing of new treatments and understanding their influence on disease progression (Brendel et al. 2016; Brendel et al. 2018; Sabri et al. 2015; Blume et al. 2018; Rominger et al. 2013; Sacher et al. 2019). Though the influence of molar activity in amyloid imaging has not been widely reported, in general preclinical imaging in small animal models (e.g. mice) requires tracers with high molar activity (Kung and Kung 2005; Jagoda et al. 2004; Sergeev et al. 2018). There seem to be mixed reports about the importance of molar activity in small animal amyloid imaging (Snellman et al. 2014), but it has been reported that detection of relatively immature (small and diffuse) amyloid lesions in mouse brain (with [^11^C]PIB) is especially sensitive to molar activity (Maeda et al. 2007).

Further development and use of these tracers in a preclinical context is hindered by limited access and/or high cost of tracers such as [^18^F]Florbetaben. Current methods and reagent kits are optimized for large-scale production, making economical production of small batches not possible with current radiosynthesizer technologies. To address these concerns, we sought to optimize production of [^18^F]Florbetaben at the microliter scale while providing high-quality product suitable for in vivo preclinical applications. Due to the small physical size of microfluidic radiosynthesizers, very low amounts of reagents and radionuclide are needed, reducing the cost of materials and equipment, and required radiation shielding (Dooraghi et al. 2013; Elizarov et al. 2010; Lebedev et al. 2012; Keng et al. 2016; Wang et al. 2017). The synthesis of multiple tracers and prosthetic groups have been successfully implemented in microliter droplet-reactor format, including [^18^F]FDG (Wang et al. 2017; Keng et al. 2012), [^18^F]Fallypride (Wang et al. 2017; Javed et al. 2014; Wang et al. 2019a), [^18^F]FET (Lisova et al. 2019), [^18^F]FDOPA (Wang et al. 2019b), [^18^F]AMBF_3_-TATE (Lisova et al. 2018), [^18^F]SFB (Kim et al. 2019) and sulfonyl [^18^F]fluoride (Fiel et al. 2015), providing a strong suggestion that [^18^F]Florbetaben could also be implemented.

To date there are no reported methods (droplet or other formats) for microfluidic synthesis of [^18^F]Florbetaben. Synthesis of [^18^F]Florbetaben via conventional methods typically follows a 2-step synthesis using an N-Boc-protected precursor (Zhang et al. 2005; Rominger et al. 2013; Patt et al. 2010; Wang et al. 2013), followed by semi-preparative HPLC purification and reformulation via C18 solid-phase extraction (SPE) (Rominger et al. 2013; Patt et al. 2010; Rowe et al. 2008). Here we develop an automated microliter droplet-based synthesis of this probe and demonstrate the benefits of using small scale production. The synthesis is performed in a microdroplet reactor, with purification via analytical-scale HPLC, and formulation carried out on a system built in-house for small-volume SPE. The low-cost approach presented here uses tiny amounts of reagents and achieves high molar activity without the need for high starting activity (as required in traditional radiosynthesizers), producing high-quality [^18^F]Florbetaben readily applicable for small animal imaging and with potential of scaling up to clinical doses.

## Materials and methods

### Reagents

No-carrier-added [^18^F]fluoride was produced by the ^18^O(p, n)^18^F reaction from [^18^O]H_2_O (84% isotopic purity, Zevacor Pharma, Noblesville, IN, USA) in an RDS-112 cyclotron (Siemens; Knoxville, TN, USA) at 11 MeV using a 1 mL tantalum target with havar foil. Acetonitrile (MeCN; anhydrous, 99.8%), methanol (MeOH; anhydrous, 99.8%), ethanol (EtOH; 200 proof, > 99.5%), hydrochloric acid (HCl; 1 M), dimethylsulfoxide (DMSO, 98%), deionized (DI) water, and polyethylene glycol 400 (PEG 400) were purchased from Millipore Sigma (St. Louis, MO, USA). All reagents were used as received without further purification. N-Boc protected mesylate FBB precursor ([methanesulfonic acid 2-{2-[2-(4-{2-[4-(tert-butoxycarbonyl-methyl-amino)-phenyl]-vinyl}-phenoxy)-ethoxy]-ethoxy}-ethyl ester) and FBB reference standard (4-[(E)-2-(4-{2-[2-(2-[^18^F]fluoroethoxy) ethoxy] ethoxy} phenyl) vinyl]-N-methylaniline) were generously provided by Life Molecular Imaging GmbH as a part of [^18^F]Florbetaben synthesis kits. Kryptofix 222 (K_222_) and potassium carbonate (K_2_CO_3_) were purchased from ABX GmbH (Radeberg, Germany). Sodium phosphate dibasic (Na_2_HPO_4_·7H_2_O) and sodium phosphate monobasic (NaH_2_PO_4_·H_2_O) were purchased from Fisher Scientific (Thermo Fisher Scientific, Waltham, MA). Ultrapure 18 MΩ water was acquired through a Milli-Q Integral 3 purification system (Millipore Sigma, St. Louis, MO, USA).

Dry scavenger mix (used in multiple steps of the reaction), consisting of sodium ascorbate with L-ascorbic acid (87:13 w/w), was obtained from the [^18^F]Florbetaben production kits provided by Life Molecular Imaging GmbH. HPLC mobile phase was prepared by first dissolving 1.785 g of Na_2_HPO_4_·7H_2_O and 0.461 g of NaH_2_PO_4_·H_2_O in 0.40 L of 18 MΩ H_2_O to make 25 mM phosphate buffer (pH 7.2), then adding in 0.60 L of MeCN. Collection mixture to recover the crude [^18^F]Florbetaben from the chip consisted of MeCN mixed with 33 mg/mL dry scavenger mix in DI water (1:1, v/v). Stabilization / dilution solution for formulation contained 39 mg/mL of dry scavenger mix in a 4:13 (v/v) mixture of PEG 400 and DI water.

### Analytical methods

A calibrated ion chamber (CRC 25-PET, Capintec, Florham Park, NJ, USA) was used to perform radioactivity measurements. For radio-thin-layer chromatography (radio-TLC) analysis, reverse phase TLC plates (RP-18 silica gel 60 F254 sheets; aluminum backing; Millipore Sigma, St. Louis, MO, USA) were cut into 15 × 60 mm pieces (with 40 mm developing distance), spotted with 1 μL of the sample and developed in 90% (v/v) MeCN in H_2_O. TLC plates were analyzed with a Cerenkov luminescence imaging system as previously described (Wang et al. 2020) or a conventional radio-TLC scanner (miniGita star, Raytest, Inc., Wilmington, NC, USA). Retention factors of the observed radioactive species were: 0.0 ([^18^F]fluoride), 0.4 ([^18^F]FBB), and 0.8 (fluorinated intermediate).

Radio-HPLC analysis (and purification) were performed on an analytical-scale Smartline HPLC system (Knauer, Berlin, Germany) with 200 μL injection loop, a pump (Model 1000), degasser (Model 5050), UV detector (Model 2500) and a radiometric detector (Bioscan B-FC-4000, Bioscan Inc., Washington DC, USA). Samples were separated using a C18 column (Luna, 5 μm particles, 100 Å pores, 250 × 4.6 mm, Phenomenex, Torrance, CA, USA) with guard column (SecurityGuard C18, Phenomenex). UV absorbance was measured at 254 nm. Using isocratic conditions with a MeCN: 25 mM phosphate buffer 60:40 (v/v) mobile phase delivered at 1.5 mL/min, the observed retention time of [^18^F]fluoride was between 2 and 3 min, 6 min for [^18^F]FBB, and 14 min for the fluorinated intermediate.

### Droplet synthesis platform

Radiochemistry was performed in droplet format using Teflon-coated silicon chips that had small circular regions of Teflon etched away, leaving hydrophilic patches that act as surface-tension traps to confine reagents during the multi-step radiosynthesis. Temperature control was achieved by affixing the chip atop a ceramic heater with thermal paste. The details of the chip fabrication were previously reported (Wang et al. 2017). Initially, the conditions were optimized manually using chips containing 4 reaction sites (Rios et al. 2019) on a platform with 4 heaters. Based on optimized conditions, the synthesis was adapted onto an ultra-compact automated droplet radiosynthesizer (Wang et al. 2019a) allowing for reduced radiation exposure and operation time via automation.

**Fig. 1:**
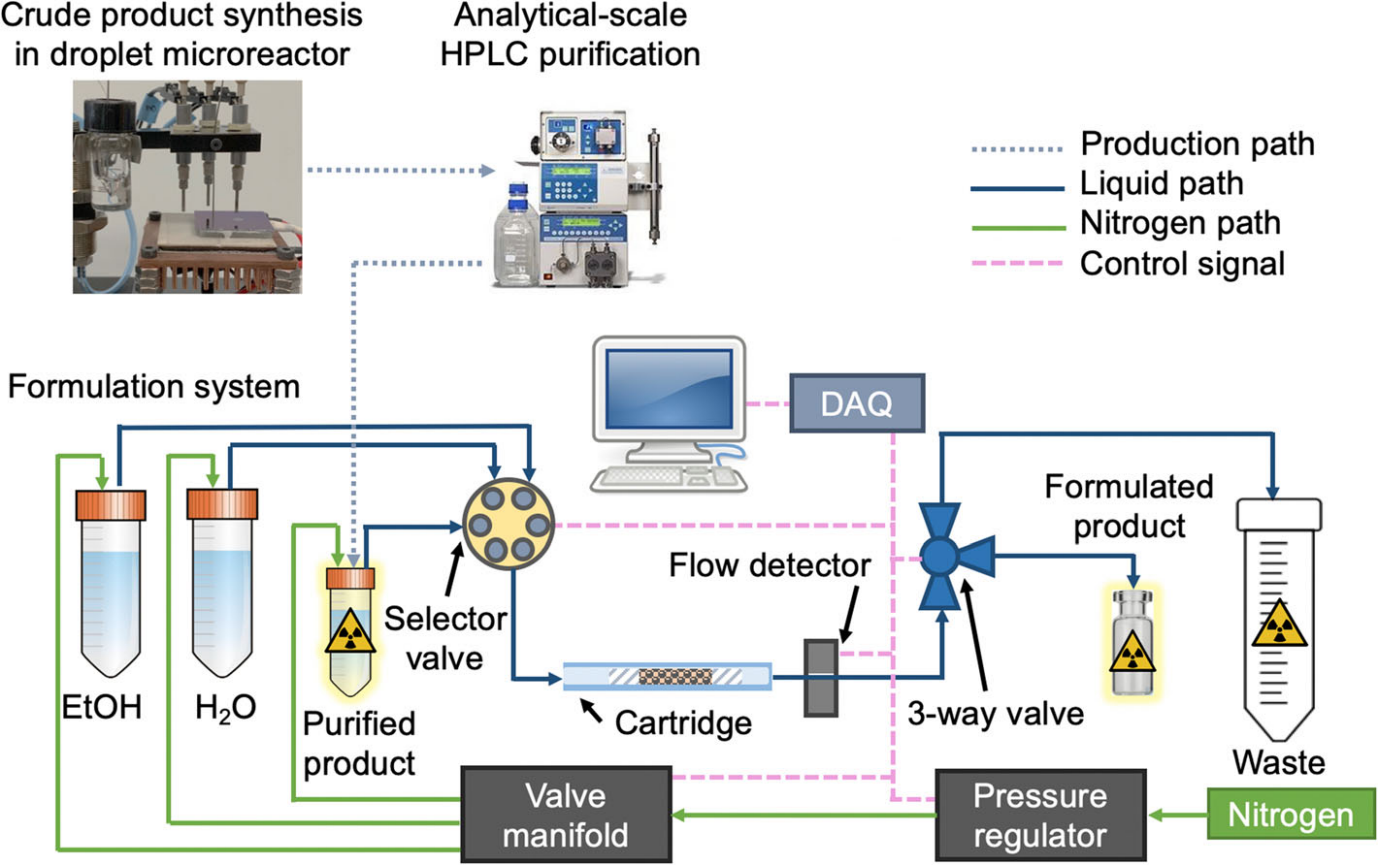
Overview of microvolume synthesis procedure for [^18^F]FBB, with detailed schematic of the formulation system (bottom)

The overall setup comprises a droplet synthesizer, analytical-scale HPLC purification, and a newly-developed automated solid-phase extraction setup to perform formulation using custom micro-cartridges with C18 resin (Fig. 1). Details of the formulation system and cartridge fabrication are provided in the Supporting information, sections 1 and 2, respectively. Briefly, the inlet of the cartridge was connected to a selector valve and the outlet to a 3-way valve. Using the selector valve, different solutions could be flowed through the cartridge such as the [^18^F]FBB fraction vial (trapping step), a vial with aqueous sodium ascorbate solution (washing step), and a vial with ethanol (elution step). The 3-way valve was used to direct the cartridge output to waste (trapping and washing steps) or the product vial (elution step). The liquid movement was initiated by applying nitrogen pressure to the vials containing [^18^F]FBB (15 psi), water (15 psi) and ethanol (3 psi). A program written in LabView (National Instruments, Austin, TX) automatically controlled the valves and pressure sources via a data acquisition module (DAQ) to complete the trapping, washing, and elution steps.

### Microvolume radiosynthesis

#### Preliminary synthesis optimization

**Fig. 2:**
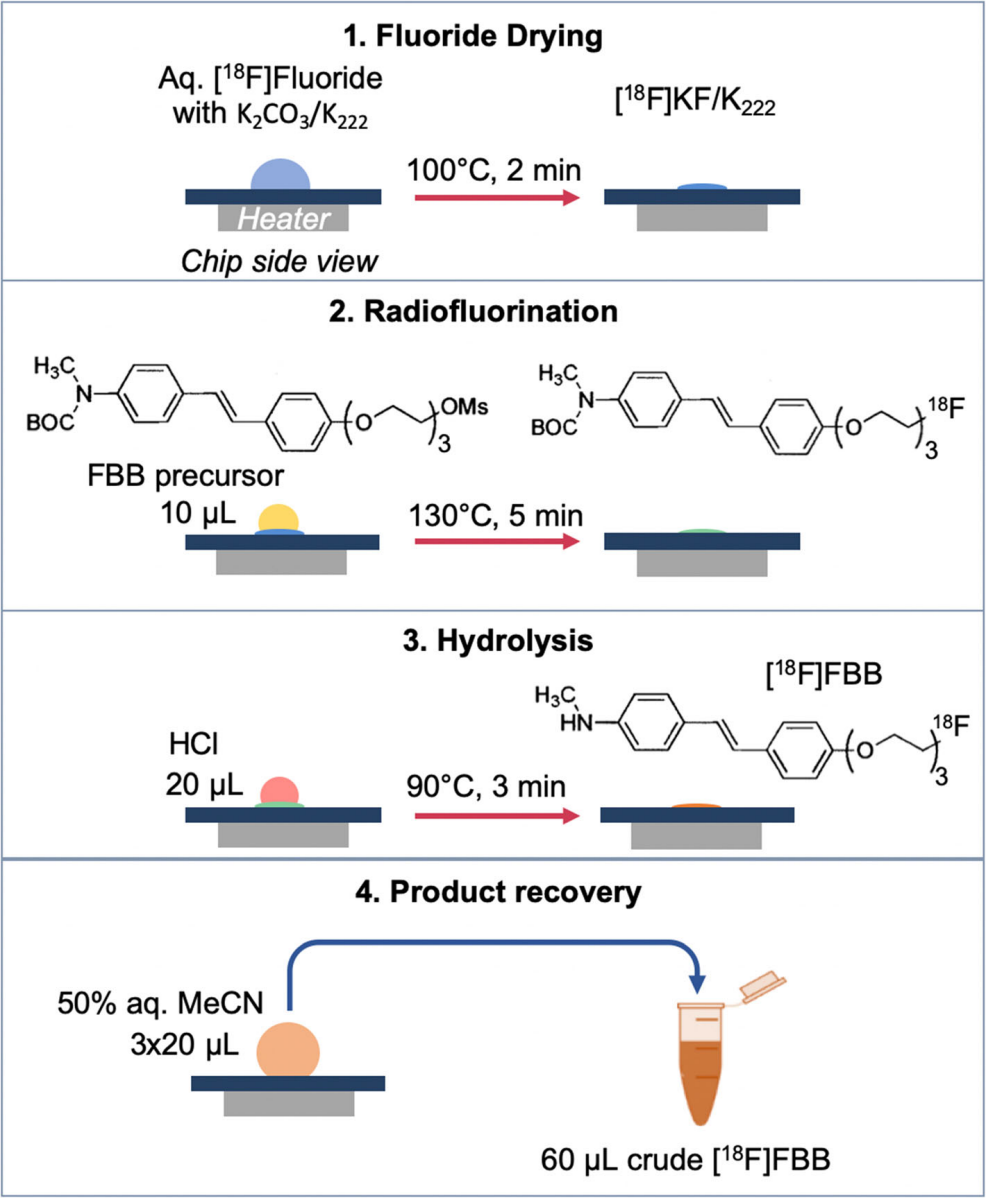
Schematic of the microscale synthesis of crude [^18^F]FBB

The microvolume synthesis was adapted from the common 2-step approach, which consists of fluorination of the Boc-protected precursor using [^18^F]KF/K_222_, followed by a hydrolysis step (Zhang et al. 2005). A schematic representation of the microvolume synthesis process is shown in Fig. 2.

Initial pre-optimization conditions for microvolume synthesis were selected by scaling down the conventional synthesis conditions reported in Collins et al. *(*Collins et al. 2017*)*. The fluorination reaction volume was reduced 90-fold from 1.8 mL to 20 μL, but the precursor concentration was maintained at 4 mM, resulting in a precursor amount of 120 nmol. The fluorination solvent was changed from MeCN to DMSO, since MeCN evaporated too quickly in droplet format. The total amount of cryptand phase-transfer catalyst was reduced 180-fold (from 49 μmol to 275 nmol of K_2_CO_3_ and from 68 μmol to 383 nmol of K_222_). First, the effect of temperature on the fluorination reaction was studied, followed by optimization of the amount of K_2_CO_3_/K_222_, and then amount of precursor. Each set of conditions was repeated *n* = 4 times, with reagents delivered manually via pipette.

For each experiment, aqueous [^18^F]fluoride (10–20 μL; ~ 7.4–370 MBq) was mixed with the desired amount of K_222_/K_2_CO_3_ in 4.5 μL H_2_O and loaded to the reaction site to be evaporated to dryness at 100 °C for 2 min. Next the desired amount of precursor in DMSO was added to the dried fluoride residue and reacted at the desired temperature for 5 min. For initial optimization experiments the crude product of the fluorination reaction was collected and analyzed. In other cases, the hydrolysis step was performed by adding 20 μL of 1 N HCl to the reaction mixture and heating at 90 °C for 3 mins. To recover the crude product (or intermediate), 20 μL of collection mixture was added to resuspend the product on chip, and then transferred into the crude product vial. To ensure thorough recovery from the chip, the collection procedure was repeated 2 more times (3 more times for the automated setup). To avoid radiolysis and photodegradation, the crude product vial was preloaded with 64 μL of water with 33 mg/mL of dry scavenger mix and kept in the dark.

#### Automated microvolume synthesis

The automated synthesis of [^18^F]FBB was performed on identical chips, but using a custom-built platform (Wang et al. 2019a) that supported automated reagent dispensing and product recovery. The reaction conditions were identical to the optimized manual synthesis conditions, except that the deprotection was performed using 1 M HCl:MeCN 1:1 (v/v). This 20 μL acidic mixture was dispensed at the beginning of deprotection and another 20 μL after 1.5 min. The diluted acid was used to reduce damage to the reagent dispensers.

### Purification and formulation

To perform purification, the crude product collected in aqueous scavenger solution was diluted with aqueous sodium phosphate buffer to a total volume of 175 μL and delivered into an analytical radio-HPLC system with 200 μL injection loop, and separated as described in the “Analytical methods” section. The [^18^F]FBB peak (retention time 6 min) was collected (for 1.0–1.5 min) into a 50 mL conical tube (Falcon, Corning, USA) pre-loaded with 33 mg/mL dry scavenger mix in 3 mL water and covered by aluminum foil.

Formulation was performed by diluting the purified [^18^F]FBB with 30 mL of DI water, and carrying out solid phase extraction (SPE) using a miniature C18 cartridge made by packing C18 resin (5 or 10 mg) into lengths of tubing (Supporting Information, Section 1). Preconditioning of miniature cartridges was performed with 5 mL MeOH followed by 6 mL of DI water at approximately 1 mL/min. An automated solid-phase extraction setup was built to perform the formulation step (Fig. 1). Details of this setup can be found in the Supporting Information, Section 2. In the final formulation procedure, the diluted [^18^F]FBB was trapped on the cartridge, and then the cartridge was washed by flowing through 10 mL DI water containing 10 mg/mL of dry scavenger mix to remove residual solvents and impurities. (The amount of scavenger is the same as reported by Rominger et al. (Rominger et al. 2013)). Finally, the trapped [^18^F]FBB was eluted from the cartridge using 150 μL EtOH into an amber-colored glass product vial preloaded with 850 μL of stabilization solution.

### Evaluation of synthesis performance

Multiple measurements were collected during the synthesis to calculate several parameters related to the synthesis performance. Unless otherwise specified, all reported percentage values (yields, efficiencies) are decay-corrected (d.c.). Starting activity was determined by calculating a difference in activity measurements of a source vial before and after addition of the radionuclide from the vial to the chip (accounting for losses in pipette tips). Collection efficiency is the ratio of activity of the crude reaction mixture recovered from the chip relative to the starting activity. Residual chip activity is the percentage of starting activity that remained on the chip after the synthesis and crude reaction mixture recovery. Radiochemical conversion is the percentage of the desired product ([^18^F]FBB) in the crude mixture as determined by radio-TLC. Crude [^18^F]FBB radiochemical yield (crude RCY) is calculated by multiplying the collection efficiency by the radiochemical conversion. Isolated yield is the ratio of the activity of the purified product collected after HPLC purification to the starting activity. Formulated product yield is calculated by dividing the activity of the final formulated product by the starting activity.

To carefully evaluate the formulation performance, additional parameters were calculated relative to the activity of pure [^18^F]FBB fraction obtained after HPLC purification. The formulation efficiency is the ratio of formulated product activity to the activity of the [^18^F]FBB fraction. Activity in waste is the ratio of activity in the waste container relative to the [^18^F]FBB fraction, and fraction collection vial residual activity is the percentage of activity remaining in the initial [^18^F]FBB fraction vial after the formulation process is complete. Cartridge residual activity is the percentage of the initial [^18^F]FBB activity that remained on the cartridge after formulation. Residual in the system is the percentage of the initial [^18^F]FBB fraction that was not recovered (i.e. remaining in various portions of the formulation system, e.g. valves, tubing, etc.).

## Results

### Microvolume synthesis optimization

**Fig. 3:**
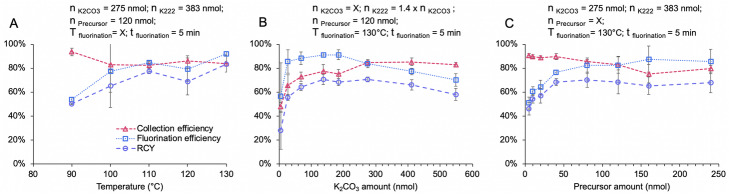
Optimization summary of fluorination step. For all reactions, [^18^F]Fluoride (aq) was evaporated to dryness with 4.5 μL of base and phase-transfer catalyst in DI H_2_O, then reacted with 20 μL precursor in DMSO for 5 min. The resulting product was analyzed via radio-TLC. **a** Effect of temperature on reaction performance; **b** Effect of base amount on the reaction (K_222_ amount is 1.4x that of K_2_CO_3_); **c** Effect of precursor concentration on the reaction. Data points represent the average of *n* = 4 repeat measurements, and error bars represent standard deviations. In each panel, the changing variable is listed with an “X” (above the graphs), and the fixed values of all other parameters are as indicated. The legend from A applies to all panels

The influence of fluorination temperature, base amount, and precursor concentration on the fluorination step (as measured by the resulting amount of fluorinated intermediate) is summarized in Fig. 3. Fluorination efficiency increased as temperature was increased from 90 to 130 °C, and while there was a very slight reduction in collection efficiency over this temperature range, the overall RCY of the intermediate increased with increasing temperature (Fig. 3a). We thus chose 130 °C as the optimal reaction temperature. The amount of base had a more complex influence on reaction performance (Fig. 3b). At low base amount (< 70 nmol K_2_CO_3_ / 100 nmol K_222_), the collection efficiency, fluorination efficiency and RCY of the intermediate increased strongly with base amount, while at moderate amounts (70–280 nmol K_2_CO_3_ / 100–390 nmol K_222_), there was relatively little change and the RCY remained nearly constant. Higher amounts of base led to a gradual reduction in fluorination efficiency and RCY of the intermediate (though collection efficiency was constant). Based on this data, the optimal base quantity was 275 nmol K_2_CO_3_ / 383 nmol K_222_. For low precursor amount (< 40 nmol), there was a rapid increase in fluorination efficiency (and RCY of the intermediate) as precursor amount was increased, and at higher amounts, the slight decrease in collection efficiency canceled out the slight increase in fluorination efficiency resulting in nearly constant RCY of the intermediate (Fig. 3c). We chose 80 nmol of precursor as the optimum value. Using the optimal conditions together, the RCY of the fluorinated intermediate (without deprotection) was 70 ± 6% (*n* = 4).

It was observed that there was a residual volume of DMSO left at the end of the fluorination reaction. Concerned that this DMSO would adversely affect downstream purification on the analytical scale HPLC (as observed previously in our lab) and would dilute the deprotection solution, we opted later to reduce the reaction volume to 10 μL. Based on our optimization data seen in Fig. 3, we did not expect much change in performance by this effective doubling of base and precursor concentrations.

After this change, the amount of residual DMSO was reduced, and after deprotection with 20 μL of 1 M HCl, the crude RCY of [^18^F]FBB was 63 ± 6% (*n* = 4) (i.e., 66 ± 6% collection efficiency and 96 ± 1% radiochemical conversion). When the synthesis was automated and performed at similar activity levels (~ 7.4 MBq starting activity), we observed a crude RCY of 58 ± 7% (*n* = 5) (i.e., 69 ± 9% collection efficiency, 86 ± 9% radiochemical conversion).

To produce quantities compatible with preclinical applications, further experiments were carried out with ~ 300 MBq starting activity level and the results are summarized in Table 1. Using the manual procedure for crude synthesis, the crude RCY was 78 ± 6 (*n* = 3) (i.e. 87 ± 1% collection efficiency and 88 ± 6% radiochemical conversion). The automated synthesis with ~ 300 MBq starting activity resulted in 60 ± 5% (*n* = 3) crude RCY (83 ± 3% collection efficiency, 72 ± 4% radiochemical conversion).

**Table 1:** Summary of synthesis performance when performed manually or on the automated setup. All % values are calculated in reference to starting activity.

	Manual Synthesis	Automated Synthesis
	*n* = 3	*n* = 3
Starting activity (MBq)	260 ± 100	330 ± 120
Collection efficiency (%)	87 ± 1	83 ± 3
Residual chip activity (%)	0.4 ± 0.2	1.2 ± 0.3
Radiochemical conversion of the crude (%)	88 ± 6	72 ± 4
Crude [^18^F]FBB yield (%)	78 ± 6	60 ± 5
Crude synthesis time (min)	30 ± 8	28 ± 1
Isolated [^18^F]FBB yield (%)	61 ± 2	53 ± 8
Molar activity d.c.^a^ (GBq/μmol)	370 ± 60	260 ± 80

^a^Molar activity is reported at the end of HPLC purification

### Purification and formulation

#### Purification

The crude [^18^F]FBB was purified using isocratic analytical scale radio-HPLC. The product was collected in ~ 3 mL volume, taking ~ 12 min for purification. In manual runs with ~ 300 MBq of starting activity, the isolated yield after purification was 61 ± 2% (*n* = 3) with molar activity of 370 ± 60 GBq/μmol decay-corrected to the end of purification (Table 1). Similarly, the equivalent experiments using automated procedure of the crude synthesis resulted in 53 ± 8% (*n* = 3) isolated yield and 260 ± 80 GBq/μmol molar activity.

#### Formulation

To ensure sufficient activity concentration of the formulated [^18^F]FBB, we developed custom-made miniature C18 cartridges with small bed volumes that could be eluted with a significantly reduced amount of EtOH compared to conventional C18 Sep-pak cartridges. To assess trapping capacity during preliminary runs of the formulation protocol, we used 5 nmol of FBB reference standard spiked with small amount of purified [^18^F]FBB (15 MBq) to simulate moderate activity levels. (In syntheses starting with ~ 370 MBq activity, we determined from the HPLC purification chromatogram that the mass of FBB was 0.7 ± 0.3 nmol (*n* = 3), therefore addition of 5 nmol would roughly correspond to a ~ 2.6 GBq batch of [^18^F]FBB.) When performing trapping with 5 mg resin only 77% of the product activity was trapped on the cartridge and 19% of the activity was found in waste (untrapped), and 77% of the initial [^18^F]FBB was recovered at the end of elution. However, trapping was more efficient for the 10 mg resin cartridge with only 1 ± 1% (*n* = 3) lost in waste (untrapped), and the elution recovered 91 ± 5% (n = 3) of the initial [^18^F]FBB. Results are summarized in Table 2. Due to the nearly quantitative trapping performance, 10 mg cartridges were used for the remaining experiments.

**Table 2:** Summary of performance of the formulation step. All % values are calculated in reference to starting pure [^18^F]FBB activity

	Manual Formulation^a^	Manual Formulation^a^	Automated Formulation
Mass of C18 resin in cartridge (mg)	5	10	10
Number of repeats	*n* = 1	*n* = 3	*n* = 3
Starting pure [^18^F]FBB activity (MBq)	7.4	15 ± 11	112 ± 51
Formulation efficiency (%)	77	91 ± 5	81 ± 5
Formulation time (min)	18	22	17
**Losses:**			
Activity in waste (%)	19	1 ± 1	3 ± 3
Fraction collection vial residual activity (%)	3	6 ± 4	2 ± 1
Cartridge residual activity (%)	0	0 ± 1	1 ± 1
Residual in the system (%)	N/A	N/A	14 ± 4

^a^Initial manual formulation runs were performed using 5 nmol of FBB spiked with purified [^18^F]FBB product, to mimic the mass loading of a higher activity level, while working with less radioactivity

These experiments were repeated using the automated formulation system (with 10 mg cartridge), resulting in 81 ± 5% (*n* = 3) recovery of the initial pure [^18^F]FBB using 150 μL EtOH, which was collected into 850 μL of stabilization solution. After 25 min formulation of 110 ± 50 MBq (*n* = 3) of activity, there was 1 mL of 77 ± 35 MBq (*n* = 3) of injection ready product. Minor activity losses were measured 3 ± 3% (*n* = 3) in waste after trapping and washing steps, and only 1 ± 1% remained on the cartridge, however it was observed that 14 ± 4% of the initial activity remained unaccounted for within the formulation system components (Table 2). Residual activity in the formulation system components was difficult to accurately measure, but substantial portions were found in the fluid path used in trapping. In the future it can be possible to perform additional rinsing step to improve product recovery.

In summary, after total of 55 min synthesis time (30 for crude synthesis, 8 min for purification and 17 min for formulation) the radiochemically pure (> 98%) [^18^F]FBB was acquired in 49 ± 3% (*n* = 3) yield with measured 340 ± 60 GBq/μmol molar activity at the end of synthesis.

## Discussion

This work shows that the synthesis of [^18^F]FBB can be performed in a microvolume reactor in relatively short time and with excellent yield and molar activity.

### High-throughput optimization

Initial optimization of the reaction was performed using high-throughput techniques (i.e., multiple reactions in parallel) (Rios et al. 2019), allowing the study of multiple reaction parameters with a high number of replicates. Notably, testing 21 sets of conditions (altering 3 parameters) with *n* = 4 repeats for each condition (a total of 84 experiments) was performed in only 6 radiochemistry sessions (12 h). Optimization of each parameter was performed sequentially in a one variable at a time (OVAT) fashion, and it is possible that additional iterations of optimization using parameters from the previous round, or more efficient and comprehensive optimization approaches such as design of experiments (DoE), could yield slight improvements, but this was not explored here.

Having extensive data about the overall impact of each parameter can be used to increase the robustness of a synthesis, i.e. by choosing parameter values where the performance is insensitive to variances in the variable (close to horizontal slope). While our optimization focused on yield, the data could also be used to optimize other outcomes, for example, minimizing the amount of precursor (to minimize cost or simplify purification) while achieving an acceptable yield.

### Purification and formulation

In this work purification was performed via analytical scale HPLC, which has been previously reported for purification of tracers produced in microfluidic systems (Lebedev et al. 2012; Wang et al. 2017; Javed et al. 2014; Wang et al. 2019a; Lisova et al. 2019; Wang et al. 2019b). Analytical-scale purification allows very short purification times (several min) and small volume (~ 1–2 mL) of pure fraction. The simplest purification and formulation route for [^18^F]Florbetaben (and the one recommended for the Life Molecular Imaging kits) would be purification via ethanolic mobile phase (sodium ascorbate buffer:EtOH, 40:60, v/v) such that the collected fraction can be formulated simply by dilution. However, when scaling to an analytical HPLC system, the pressure limit was exceeded for amounts of EtOH > 20% (v/v) at 1 mL/min, and the late retention time of [^18^F]FBB when using lower amounts of EtOH was not practical. It is possible that other column types or sizes could avoid this problem, but we instead used an MeCN-based mobile phase for separation, which required a downstream formulation step to remove the acetonitrile after purification. Under this condition, the crude product was rapidly and efficiently purified and collected within 8 min of injecting the crude reaction mixture.

Initially, we tried evaporative solvent exchange to remove MeCN, performing evaporation at 100 °C followed by resuspension of the dried product in PBS. However, evaporation of 2–3 mL of volume of collected crude product took a significant amount of time (15 min). We also observed the solution change from clear to cloudy, and after resuspension in formulation buffer, a significant amount of product (~ 70% of initial pure [^18^F]FBB) was stuck to the vial, likely due to the poor solubility of this compound.

Instead, formulation via solid-phase extraction was explored using a C18 cartridge as widely reported by others (Table 3) (Rominger et al. 2013; Patt et al. 2010; Rowe et al. 2008). However, commercial C18 cartridges (Waters Sep-pak C18 Plus Light, 130 mg, Waters Corporation, Milford, MA) were not suitable for small batch syntheses: to efficiently elute the product from the cartridge, 1–2 mL EtOH was needed, which would then be diluted with stabilizing solution to a total volume of 7–14 mL to lower the EtOH concentration to 15% (acceptable limit for the formulation of this probe). For preparation of small batches of [^18^F]FBB (e.g. < 185 MBq), the resulting concentration (< 26 MBq/mL) would be too dilute for small animal imaging. Instead, we explored the use of custom miniature cartridges made by packing C18 resin into lengths of tubing (Supporting Information, Section 1), which allowed minimization of the final formulated volume. Cartridges made inside 0.02″ ID tubing exhibited extremely low flow rates, but cartridges packed inside 0.0625″ ID tubing had suitable flow rates. With these miniature cartridges, the product could be efficiently recovered with only 150 μL EtOH, enabling formulation (to dilute EtOH to an acceptable level of 15%) in a final volume of 1 mL.

**Table 3:** Comparison of the performance of the microscale synthesis reported in this work to literature methods

Reference	This work	(Zhang et al. 2005)	(Rowe et al. 2008)	(Wang et al. 2011)	(Rominger et al. 2013)	(Patt et al. 2010)	(Collins et al. 2017)	(Brendel et al. 2015)
Number of replicates	*n* = 3	NR	NR	*n* = 4	NR	*n* = 10	*n* = 3	NR
Precursor amount (mg)	0.052	4	NR	5	5	NR^a^	7	5
Reaction solvent	DMSO	DMSO	NR	DMSO	MeCN	NR^a^	MeCN	MeCN
Reaction volume (mL)	0.01	0.2	NR	0.5	1	NR^a^	1.8	1
Formulated productyield (%; d.c.)	49 ± 3	30^c^	NR	23 ± 3	18	7.8 ± 2.6	60 ± 9 ^b^	18
Formulation method	SPE (micro C18 cartridge)	NR	SPE (Sep-pak C18)	SPE (Sep-pakplus C18)	SPE (Sep-paklight C18)	SPE (Strata-X 33 μm,reversed phase)	NR	SPE (Sep-pak light C18)
Synthesis time (min)	55	90^c^	60	45^d^	75	50	44^b^	75
Molar activity at EOS(GBq/μmol)	340 ± 60	48–56	170	25–30	80	220 ± 170	NR	50–90

*Abbreviations*: *NR* Not reported, *EOS* End of synthesis, *SPE* solid-phase extraction

^a^The conditions are not reported precisely, but method by Zhang et. al. is cited as a reference

^b^The reported data is up to the end of HPLC purification (excluding formulation). Yield is therefore overestimated and synthesis time underestimated

^c^The formulation procedure is not reported or discussed. Yield may be an overestimate and synthesis time an underestimate

^d^In this rapid method, purification was performed with SPE (no HPLC), but not all impurities were removed

Unfortunately, the formulation requires extra time (17 min), leading to some radioactive decay during the process. The formulation process was very efficient when performed manually, however the presence of extra fluidic components in the automated system resulted in slightly reduced efficiency. Additional losses in the automated formulation system resulted in a 10 point drop in formulation efficiency compared to manual method (i.e. 81% versus 91%).

In the current formulation protocol, the trapping step takes up the majority of time, requiring ~ 10 min in total to process nearly 30 mL of volume, while the washing and elution steps take 3 and 4 min, respectively. Possible directions for improvement would be to optimize the HPLC purification method to reduce peak width and collection volume, leading to a lower dilution volume, or optimizing the applied pressure during trapping to achieve both highest trapping efficiency and flow rate.

### Probe stability and radiolysis

It has been reported that N-methylaniline substituents can make this, and structurally similar, beta-amyloid PET tracers, quite susceptible to radiolysis (Scott et al. 2009). In fact, significant radiolysis (8 point reduction in radiochemical purity) was already observed within 10 min for [^18^F]AV-19 in isotonic saline with 5% EtOH at a modest activity concentration of 185 MBq/mL. Scott et al. found that addition of appropriate radical scavengers extended stability of the compound to multiple hours even in larger scale productions yielding 7.4 GBq of product (Scott et al. 2009).

To ensure highest radiochemical purity and stability of [^18^F]FBB produced with microvolume method, the scavenger had to be introduced at various steps of the production. Using literature reports as a guide (Rominger et al. 2013; Scott et al. 2009), scavenger was introduced into the collection solution during collection of the crude product from the chip, pre-loaded in the collection vial prior to recovery of the pure fraction from HPLC, in the dilution solution used prior to formulation, and pre-loaded in the final product vial.

### Benefits of microscale synthesis

The whole production (synthesis, purification, formulation) can be completed in 55 min with high yield (~ 50% decay-corrected). The 2-step reaction itself was quite fast, due largely to the small volume reaction which requires very little time for heating/cooling or performing solvent evaporation steps (e.g. during [^18^F]fluoride drying). Though not explored here, there is room to improve and develop a faster analytical-scale HPLC separation process, and to shorten the formulation process as discussed in detail above. It is also feasible to increase the amount of starting activity (i.e. loading larger volume of [^18^F]fluoride or using a radioisotope concentrator (Chao et al. 2018)) to produce higher amounts of the probe.

Another advantage of the microscale synthesis is that high molar activity 340 GBq/μmol could be achieved at the end of synthesis, even when starting with low amounts (< 350 MBq) of [^18^F]fluoride, something that cannot be achieved with conventional macroscale synthesis processes (Sergeev et al. 2018). Due to the importance of high molar activity when imaging certain tracers in small animals, the ability to achieve high molar activity in small batches can be a huge advantage, avoiding significant waste of radionuclide and probe that would otherwise be needed (i.e. to prepare a large batch to ensure high molar activity, but discard most of it since only a small quantity is needed for animal imaging). High molar activity of the amyloid tracer [^11^C]PIB has been reported to be particularly important for detection of relatively immature amyloid lesions in small animal models (Maeda et al. 2007), suggesting it could be critical in certain cases for [^18^F]FBB imaging as well. Additionally, high molar activity provides a longer duration over which the molar activity can be adjusted to be within a desired range for experiments, and helps to lower the total injected mass of the tracer.

In comparison with literature methods (Table 3), the yield of this microvolume synthesis is among the highest, while only consuming ~ 1% the amount of precursor as other methods. In combination with the simple apparatus and compact size, this could lead to lower costs in production of [^18^F]FBB, and potentially enable new models of production and distribution for preclinical research. The microvolume synthesis approach could also provide a way for existing production facilities to add capability for production of additional tracers without significant need for additional hot cell space.

## Conclusion

We implemented an efficient synthesis of [^18^F]FBB in microdroplet format on simple chips with surface-tension traps acting as reaction sites. The overall synthesis was fast (55 min), high-yielding (49 ± 3%, *n* = 3), and had high molar activity (340 ± 60 GBq/μmol, *n* = 3). The combination of a droplet radiosynthesizer with analytical-scale radio-HPLC purification system, and a miniature SPE-based formulation system, provides a platform for streamlined and economical production of [^18^F]FBB, and could be extended to other tracers in a straightforward manner.

## Supplementary Information

**Additional file 1.**

## Data Availability

Most of the experimental data is reported in the summary tables in the manuscript, and representative analytical chromatograms are shown in the Supplemental information. The additional datasets for each individual experiment are available from the corresponding author on reasonable request.

## Abbreviations

PET: Positron emission tomography
SPE: Solid phase extraction
TLC: Thin-layer chromatography
HPLC: High performance liquid chromatography
DI: Deionized
RCY: Radiochemical yield
D.c.: Decay-corrected
ID: Inner diameter
DAQ: Data acquisition
