# Supplementary material for: A simple and efficient automated microvolume radiosynthesis of [18F]Florbetaben
Source: EJNMMI Radiopharm Chem. 2020 Dec 4;5:30. doi: 10.1186/s41181-020-00113-w (PMC7718361; doi:10.1186/s41181-020-00113-w)
Supplement: Supplementary file 1 — Additional file 1. [file 41181_2020_113_MOESM1_ESM.pdf]

Supporting information

A simple and efficient automated microvolume radiosynthesis of [^{18}F]Florbetaben

Ksenia Lisova[1,2,3], Jia Wang[2,3,4], Philip H. Chao[2,3,4], R. Michael van Dam[1,2,3,4*]

[1]Physics in Biology and Medicine Interdepartmental Graduate Program; [2]Crump Institute for Molecular Imaging; [3]Department of Molecular & Medical Pharmacology; [4] Department of Bioengineering, University of California Los Angeles, Los Angeles, CA, USA

[*]Corresponding author:
R. Michael van Dam, Email: mvandam@mednet.ucla.edu
Address: 4323 CNSI, 570 Westwood Plaza, Los Angeles, CA 90095, USA; Phone: +1-310-206-6507

Contents

1. Fabrication of miniature C18 cartridges 1
2. Additional details of the formulation system 2
3. UV calibration for molar activity determination 3
4. Sample HPLC's 4

1 Fabrication of miniature C18 cartridges

1.1 Materials

- Perfluoroalkoxy alkane (PFA) tubing, 0.125” OD x 0.0625” ID (IDEX, Lake forest, IL)
- C18 Sep-Pak Plus Light cartridges (Waters, Milford, MA)
- Integra™ Miltex™ 1/16” standard biopsy punches (Thermo Fisher, Waltham, MA)
- Eppendorf™ 0.2mL PCR Tube (Thermo Fisher, Waltham, MA)
- Polyethylene frits, 1/8” thickness, 20 µm pore size, 75 mL size (Waters, Milford, MA).

1.2 Method

The tubing was stretched slightly (~10 % length increase) prior to using to reduce the inner diameter allowing for a tighter fit with the frits, then cut into 10 cm long pieces. One small frit was cut from the larger frit piece using a 1/16” biopsy punch and directly pushed out from the punch into one end of the tubing (the “input” side). Using a thin rod, the frit was push until it was 2.5 cm distance into the tubing.

The tubing next to the frit was gently pinched to prevent frit movement. The C18 resin was obtained by emptying a commercial C18 cartridge. The desired mass of resin (10 mg) was weighed in a PCR tube to which 200 µL of MeOH was added and stirred. To load the slurry into the tubing, the cartridge was connect to vacuum on the input side of the tubing, and the output end was dipped into the PCR tube, drawing the resin into the tubing. The PCR tube was filled with additional MEOH and vacuum was applied to ensure all resin ended up inside the tubing-cartridge. This washing was performed twice. After the resin was loaded, another frit was inserted from the "output" side of the tubing, pushed gently until it touched the resin, and then the tubing was pinched to fix all the components in place. A diagram and photograph are shown in **Figure S1**.

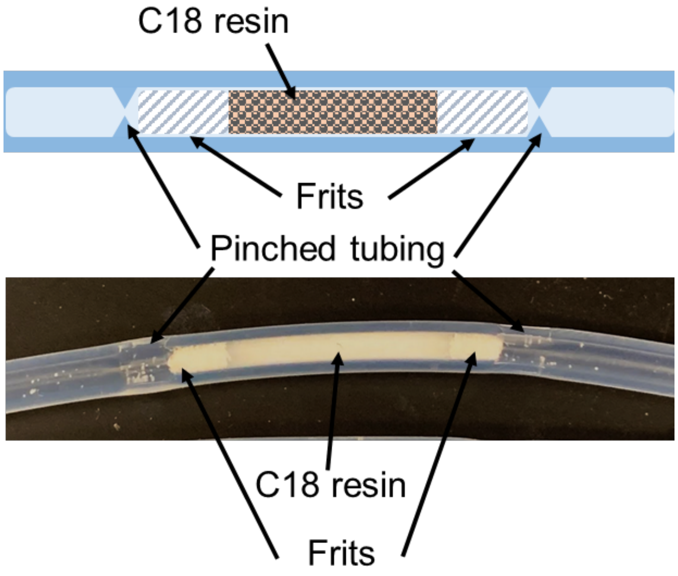

Figure S1. Schematic (above) and photograph (below) of the microscale C18 cartridge used for [^{18}F]florbetaben formulation

2 Additional details of the formulation system

PFA tubing (0.125" OD x 0.0625" ID), PFA tubing (0.0625" OD x 0.02" ID), and 1/4-28" fittings and unions (for 0.125" OD and 0.0625" OD tubing) were purchased from IDEX Corporation (Lake Forest, IL, USA). The larger tubing was used for the 3-way valve to waste line; smaller tubing was used for all other fluid connections. The Rheodyne model EV750-105 selector valve was purchased from IDEX, and the data acquisition module (DAQ) model E-1608 was purchased from Measurement computing (Norton, MA). The 3-way liquid valve model LVM105R-5C-2, pressure regulator model ITV0050-2UL (with 0.001-0.9 MPa output), 3-port solenoid valves model S070B-5DG (vacuum to ~0.5MPa), mounted on a manifold bar base (SS073-B01-03 C), were all purchased from SMC Pneumatics (SMC, Japan).

A schematic of the automated formulation system is shown in the main paper (**Figure 1**) and a photograph of the system is shown in **Figure S2**. Control of all electronic components was performed by controlling a DAQ with a custom interface created in LabView (National Instruments). The input of the pressure regulator was connected to a nitrogen source (set at 58 psi) and the output connected to 3 separate 3-way pneumatic valves for supplying pressure to the headspace of the purified product vial (for trapping step), DI water reservoir (for washing step), and EtOH reservoir (for elution step). The

analog voltage from the DAQ to the pressure regulator was calibrated to enable precise control of pressure from the LabView program. On/off positions of the 3-way liquid valve and pneumatic valves were switched by a DAQ-controlled custom Darlington board. The input of the micro-cartridge was connected via the selector valve to the product vial, DI water reservoir and EtOH reservoir, and the output of the cartridge was connected via the 3-way liquid valve to either waste or the formulated product vial.

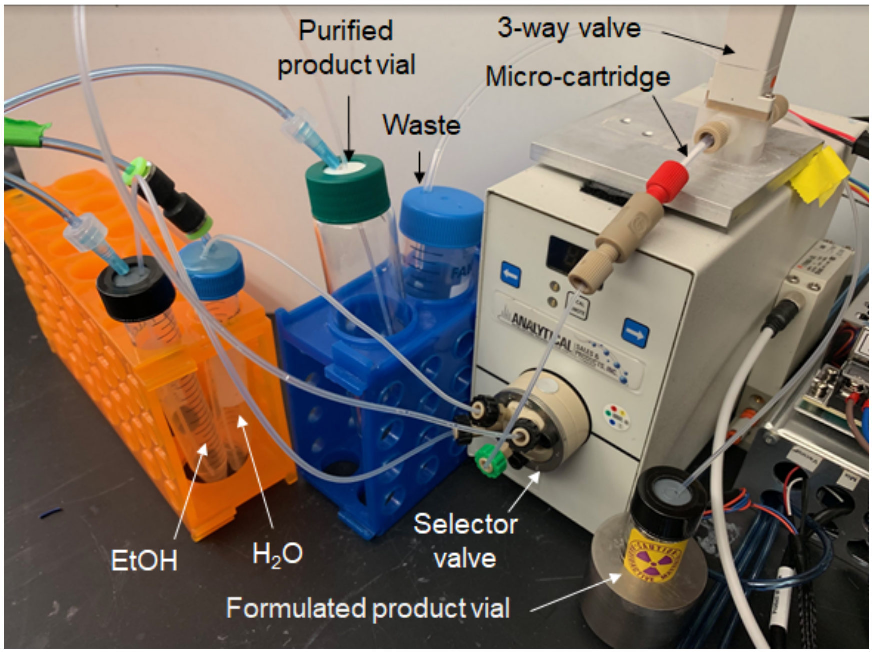

Figure S2. Photograph of the automated formulation system.

3 UV calibration for molar activity determination

The calibration curve (**Figure S3**) was created using 0.05 mM FBB reference standard in MeCN. Known quantities of the standard were injected into the analytical HPLC and the area under the curve was computed for the corresponding peak in the UV absorbance chromatogram.

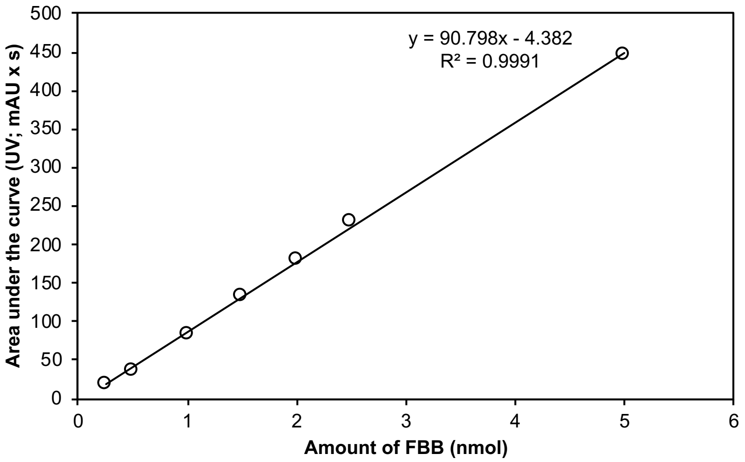

Figure S3: Calibration curve related amount of FBB injected in analytical HPLC to the area under the curve in the UV absorbance chromatogram. n = 1 for each value.

4 Example HPLC chromatograms

In all cases, samples were injected on an analytical C18 column with flow rate of 1.5 mL/min. The mobile phase for most samples was 60:40 MeCN:25 mM phosphate buffer 1:1 (v/v) ; exceptions are noted below. We present below sample chromatograms of a crude [^{18}F]FBB sample during purification (**Figure S4**), crude fluorinated intermediate (**Figure S5**), and formulated [^{18}F]FBB (**Figure S6**).

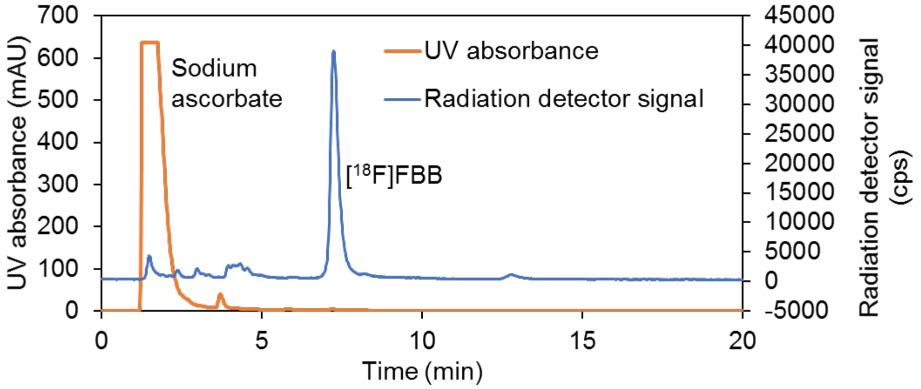

Figure S4. Example HPLC chromatogram of a crude [^{18}F]FBB sample.

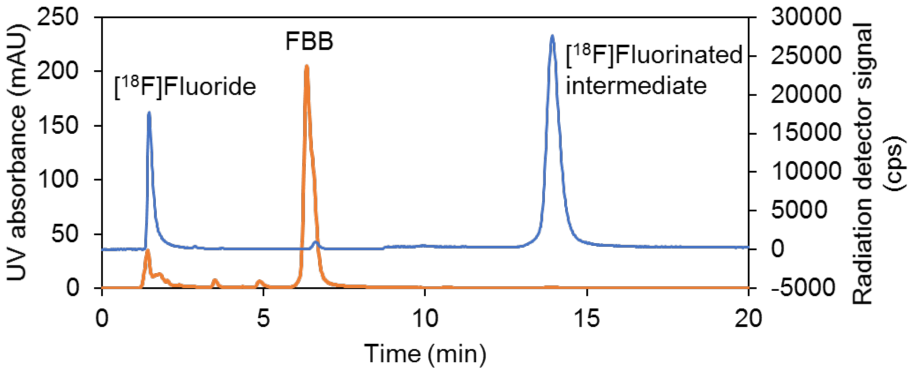

Figure S5. Example HPLC chromatogram from a crude sample of the fluorinated intermediate, co-injected with FBB reference standard.

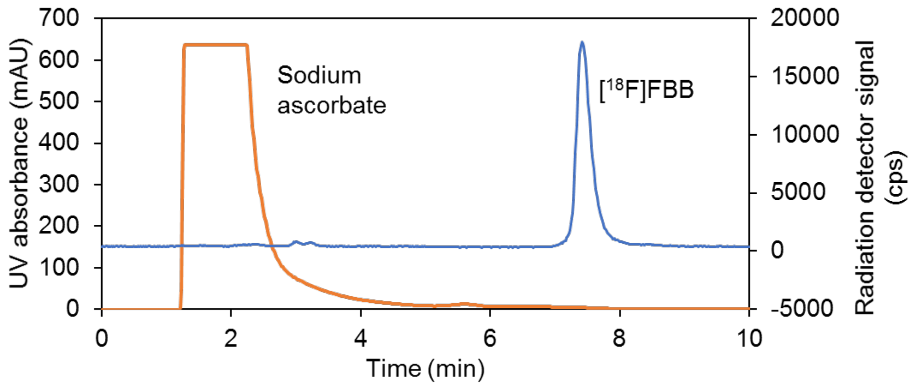

Figure S6. Example HPLC chromatogram from a formulated sample of [^{18}F]FBB (98% radiochemical purity). Note the x-axis range is different than in **Figures S4** and **S5**.

We also include below some HPLC chromatograms showing signs of radiolysis (**Figure S7**) and photodegradation (**Figure S8**).

In the chromatograms of **Figure S7**, both samples were obtained after fraction collection from HPLC and stored in amber glass vials. For the left chromatogram, a 0.2 mL (~7 MBq) aliquot was taken and diluted with 1 mL sodium ascorbate solution (33 mg/mL in DI water), and stored for 2 h. For the right chromatogram, 0.2 mL (~7 MBq) of the same purified sample was stored at room temperature in the HPLC mobile phase (no ascorbate) for 2 h.

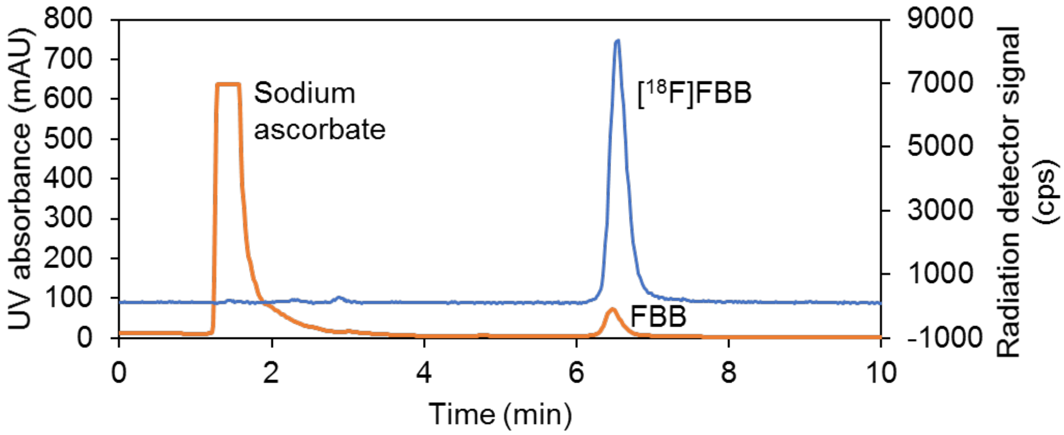

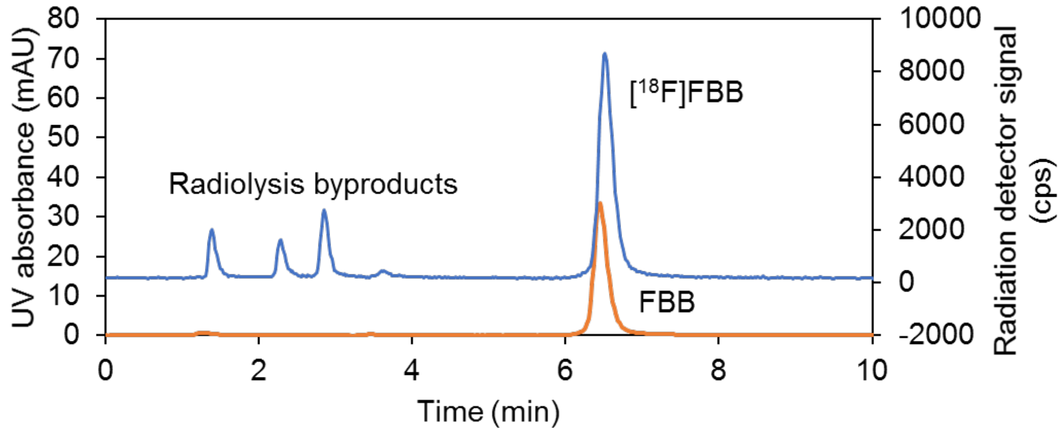

Figure S7: Example chromatograms showing sample degradation due to radiolysis. (Top) Sample after 2 h incubation in ascorbate-containing buffer (1.2 mL total volume) remained at 98% radiochemical purity. (Bottom) Sample after 2 h incubation without ascorbate in the buffer (0.2 mL volume) dropped to 72% radiochemical purity due to formation of radiolysis byproducts. Both samples were divided up from the same original batch after HPLC purification each having ~7 MBq of activity, and were mixed with FBB reference standard just prior to obtaining the HPLC chromatograms.

In **Figure S8** are shown HPLC chromatograms of FBB standard dissolved in HPLC mobile phase before and after being stored at room temperature for 1.5 hours. The upper chromatogram belongs to fresh sample at t = 0. For the middle chromatogram, the sample was stored in a transparent glass vial under room light, resulting in a secondary peak appearance after FBB peak. For the lower chromatogram, an aliquot of the same sample was stored under identical conditions except in an amber glass vial.

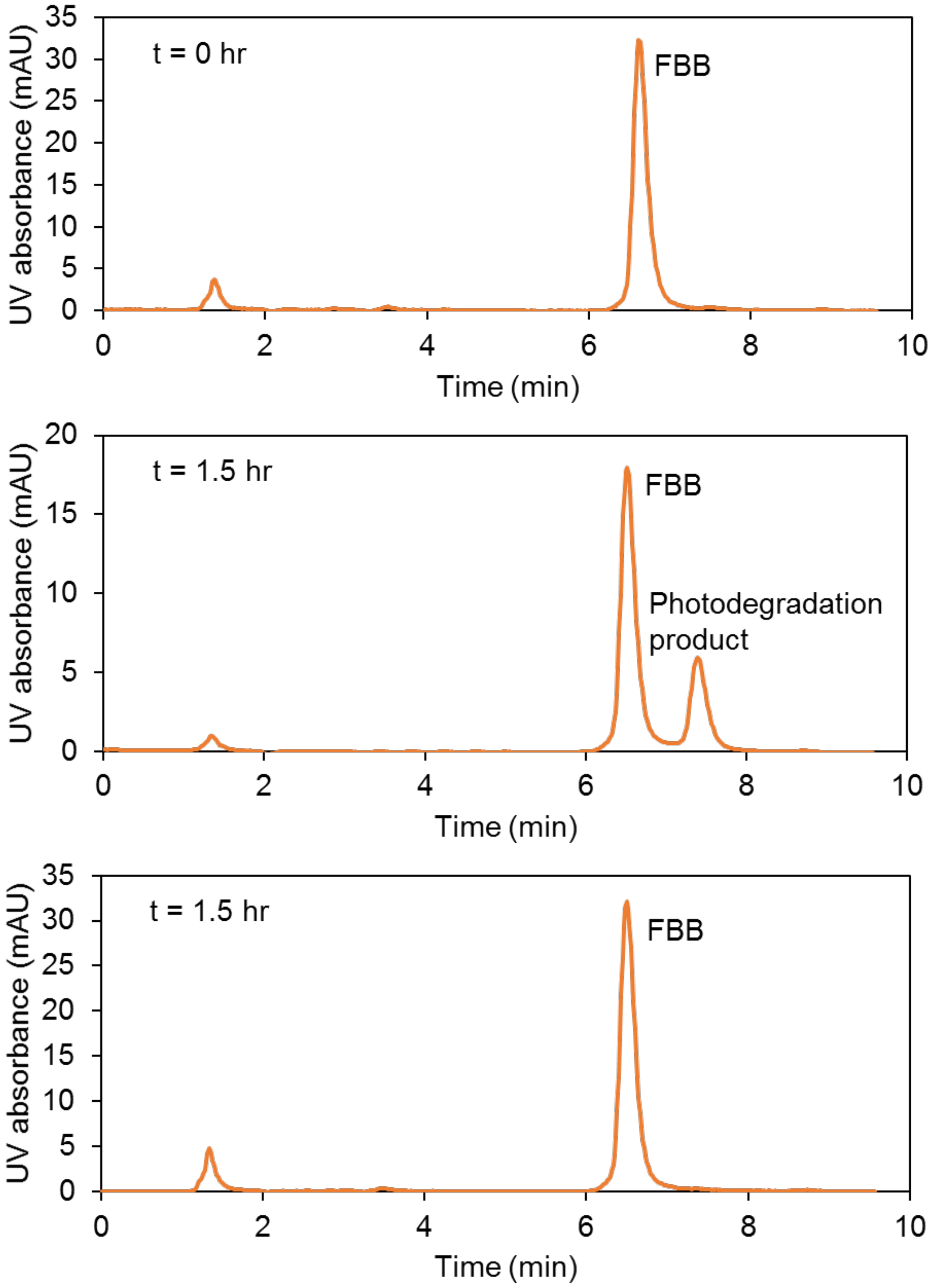

Figure S8: Example HPLC chromatograms showing evidence of photodegradation. (Top) Initial sample. (Middle) Sample stored in a transparent vial in ambient room light for 1.5 h. (Bottom) Sample stored in an amber vial for 1.5 h.
